# Effects of Organic-Loading-Rate Reduction on Sludge Biomass and Microbial Community in a Deteriorated Pilot-Scale Membrane Bioreactor

**DOI:** 10.1264/jsme2.ME16015

**Published:** 2016-07-12

**Authors:** Yuya Sato, Tomoyuki Hori, Ronald R. Navarro, Ryuichi Naganawa, Hiroshi Habe, Atsushi Ogata

**Affiliations:** 1 Environmental Management Research Institute, National Institute of Advanced Industrial Science and Technology (AIST)16–1 Onogawa, Tsukuba, Ibaraki 305–8569Japan; 2 Research Institute for Sustainable Chemistry, AIST1–1–1 Higashi, Tsukuba, Ibaraki 305–8565Japan

**Keywords:** activated sludge, microbial community, membrane bioreactor, reduction of excess sludge

## Abstract

The effects of a precipitous decrease in the inlet organic loading rate on sludge reductions and the microbial community in a membrane bioreactor were investigated. The sludge biomass was markedly reduced to 47.4% of the initial concentration (approximately 15,000 mg L^−1^) within 7 d after the organic loading rate was decreased by half (450 to 225 mg chemical oxygen demand L^−1^ d^−1^). An analysis of the microbial community structure using high-throughput sequencing revealed an increase in the abundance of facultative predatory bacteria-related operational taxonomic units as well as microorganisms tolerant to environmental stress belonging to the classes *Deinococci* and *Betaproteobacteria*.

The membrane bioreactor (MBR) process is becoming an increasingly important wastewater treatment technology because of its advantages, including a higher effluent quality and longer sludge retention time due to the actions of membrane filtration, which retains the biomass inside the reactor. Hence, this process allows for operation under higher mixed liquor suspended solid (MLSS) concentrations so that a larger organic load may be applied than that in the conventional activated sludge method (12).

One of the main drawbacks in the operation of MBRs with high MLSS concentrations is the insufficient supplementation of dissolved oxygen (DO) to activated sludge (24). An increase in the viscosity of activated sludge at high MLSS concentrations (*e.g.*, >10,000 mg L^−1^) results in inefficient oxygen dissolution, even at higher airflow rates (9, 19). This may lead to a relatively anaerobic condition, resulting in the predominance of obligatory anaerobic microorganisms in the bioreactor. These anaerobes may cause foul odors and decrease the efficiency of the oxidative degradation of organic substances, which ultimately leads to the deterioration of MBR performance.

In these cases, reductions in excess sludge are needed in order to recover MBR performance, which may improve sludge aeration and increase DO levels. So far, chemical and thermal treatments have been applied to reduce excess sludge biomass during the operation of MBRs (8, 23). We very recently reported that a decrease in the feed to microorganism ratio during a simulated rainfall condition suppressed an increase in the sludge biomass in the MBR (20). Therefore, we herein examined the effects of lowering the inlet organic loading rate (OLR) on sludge biomass reductions in a deteriorated pilot-scale MBR. Microbial community dynamics during the operation were also investigated by high-throughput sequencing of 16S rRNA genes.

The experimental set-up consisting of a pilot-scale MBR system (operating volume of 230 L and 0.24-m^2^ flat polyacrylonitrile membrane module; Awa Paper Mfg, Tokushima, Japan) and activated sludge (from Kinu-aqua station, Ibaraki, Japan), and the synthetic wastewater composition used was described in our previous studies (18, 19). The operational conditions, such as the flow rate of sludge aeration (95–100 L min^−1^), inlet synthetic wastewater, membrane-filtrated permeate, and return sludge (115 L d^−1^; hydraulic retention time=2 d), as well as the analytical methods for physicochemical parameters (including MLSS, DO, chemical oxygen demand [COD], and total organic carbon [TOC]), were also the same as in our previous studies (18, 19).

The MBR was pre-operated for more than 28 d at an OLR of 450 mg COD L^−1^ d^−1^. Between day 1 to 5 of the actual experimental run, the MBR was continuously operated at the same feed substrate concentration as pre-operation. As shown in Fig. 1A, MLSS concentrations were maintained at high levels during this initial stage (15,000–16,867 mg L^−1^). In contrast, DO levels were low (<0.2 mg L^−1^) (Fig. 1B), indicating that the sludge environment was microaerobic or partially anaerobic. An aerobic condition was difficult to achieve at the employed airflow rate of 100 mL min^−1^ under the high MLSS conditions (19). Low COD removal rates were observed during this period (73%–80%) (Fig. 1C), suggesting the deterioration of the reactor condition to some extent.

After sludge sampling and physicochemical measurements on day 5, the substrate concentration of the inlet wastewater was decreased by half (an OLR of 225 mg COD L^−1^ d^−1^), and the MBR continued to be operated until day 21. The MLSS value markedly decreased from days 5 to 15 (from 16,867 to 5,400 mg L^−1^) (Fig. 1A) in response to this operational change. In contrast, the TOC and COD values in the effluent (TOC, from 69.2 to 196 mg L^−1^; COD, from 251 to 510 mg L^−1^; Fig. 1C) did not increase as much as that observed inside the reactor (*i.e.* sludge supernatant) during this period (TOC, from approximately 500 to 1,000 mg L^−1^; figure not shown). These results indicate that the majority of cellular macromolecules released during microbial cell lysis did not permeate through the membrane. Furthermore, the DO level increased and reached 3.93 mg L^−1^ by day 15 (Fig. 1B), suggesting the restoration of an aerobic environment in the activated sludge. After day 15, the DO increased further (3.33–5.44 mg L^−1^) and the CO_2_ gas concentration measured by the gas sensor CDM4161-L00 (Figaro, Osaka, Japan) also increased (data not shown), implying the occurrence of the complete oxidative degradation of organic matter in the sludge.

Microbial population dynamics in the activated sludge were evaluated using the high-throughput Illumina sequencing of 16S rRNA genes, as we reported previously (2, 14). The total number of sequences obtained from 17 sludge samples was approximately 0.75 million, corresponding to an average of 43,949 sequences in each library. Raw sequence data in this study were deposited in the MG-RAST database (http://metagenomics.anl.gov/) as “Effects of organic loading rate reductions on a deteriorated MBR 2015” project under the IDs 4684066.3–4684082.3. Phylogenetic analyses of Illumina sequence data revealed marked changes in the microbial community composition in response to operational conditions (Fig. 2A and B). At day 5, the activated sludge was mainly predominated by *Betaproteobacteria* (particularly the genus *Acidovorax*), with a relative abundance of 58.7% (Fig. 2A). Additionally, a large portion of the activated sludge was composed of obligate anaerobic bacteria belonging to the classes *Clostridia* (11.0%) and *Bacteroidia* (17.0%). However, the abundances of *Clostridia* and *Bacteroidia* declined with a decrease in the inlet COD concentration after day 5; their relative abundances decreased to 0.042% and 0.423%, respectively, by day 15. These changes may be explained in relation to the increased DO level in the MBR (Fig. 1B). Furthermore, the population size of the genus *Acidovorax* largely decreased (Fig. 1B). In our previous studies, *Acidovorax* sp. was found to dominate sludge microbial communities under low-DO and high-OLR conditions (18, 20), implying that increased DO levels and decreased OLR are disadvantageous for this bacterium. Although the MLSS decreased to approximately one-third of its initial value in this period, the populations of several bacterial groups increased. Specifically, the relative abundance of the class *Deinococci* increased after day 5, and reached 63.3% by day 15 (Fig. 2A). Within this class, the operational taxonomic unit (OTU) related to *Truepera radiovictrix* was the most abundant (Fig. 2B, Table 1); *T. radiovictrix* is known to be capable of growth under extreme environmental conditions, for example, 0% to 7% NaCl, 25°C to 55°C, and pH 6.5 to 11.2 (1). During the operation of the reactor, the pH of the sludge increased gradually from 8.97 (day 5) to 9.42 (day 39), which may be advantageous for *Truepera* sp., allowing it to dominate the microbial population. Although the abundance of *Betaproteobacteria* remained relatively high throughout the operation of the reactor, the main constituent changed from *Acidovorax* sp. to *Parapusillimonas* sp. (Fig. 2B). *Parapusillimonas granuli* (Table 1) was originally isolated from activated sludge granules (11). Its ability to form aggregates with other cells may have rendered *P. granuli* capable of surviving during marked conditional changes.

Table 1 summarizes the top 10 OTUs with the highest increases in abundances at day 15 relative to day 5. The list emphasizes increases in several OTUs related to bacterial micro-predators. For example, *Sorangium cellulosum* is a facultative predator (17), and a *Luteimonas*-related OTU was 98% identical to *Lysobacter* sp., which is also known to be a facultative predator (22). These bacterial predators were also found in our previous studies during periods of MLSS decline (19), and, thus, may play specific roles in lysing and/or degrading microbial cells in the sludge. Besides protozoa, which are regarded as crucial predators in activated sludge (15), such bacterial predators may also contribute to reducing MLSS concentrations.

In conclusion, we herein demonstrated that a precipitous decrease in organic loading was effective in markedly reducing the sludge biomass in the deteriorated MBR. Lowering the organic substrate feed resulted in an increase in DO levels, and, subsequently, a decrease in the abundances of obligate anaerobic microorganisms such as those affiliated with *Clostridia* and *Bacteroidia*. In contrast, an increase in the abundances of facultative predatory bacteria-related OTUs was concurrently observed. Additionally, microorganisms tolerant to extreme environments or marked conditional changes appeared to become dominant. In order to obtain a more detailed understanding of the process of reducing excess sludge by controlling inlet organic loading and sludge microorganisms, further investigations of biomass reduction mechanisms, including the involvement of protozoa, are needed.

## Figures and Tables

**Fig. 1:**
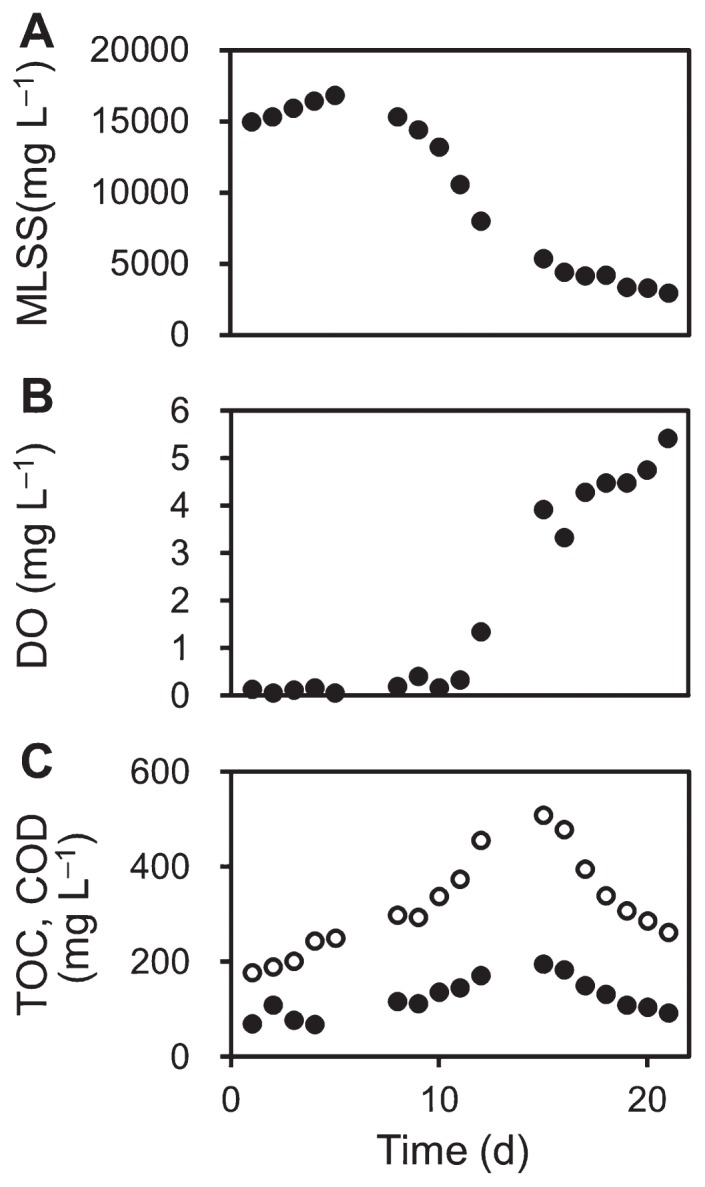
Changes in physicochemical parameters of the sludge and effluent. (A) MLSS in the sludge. (B) DO levels in the sludge. (C) Closed circles, TOC in the effluent; open circles, COD in the effluent. The construction of the pilot-scale MBR used in this study was described in our previous studies (18, 19). TOC levels in the effluent were determined using a TOC analyzer (TOC-L; Shimadzu, Kyoto, Japan). COD levels in the effluent were measured with a COD analyzer (DR2800 and DRB200; Hach, Loveland, CO, USA) using appropriate kits (TNT820 or TNT821, Hach). Data for MLSS and DO are presented as mean values from three different sampling points of the reactor.

**Fig. 2:**
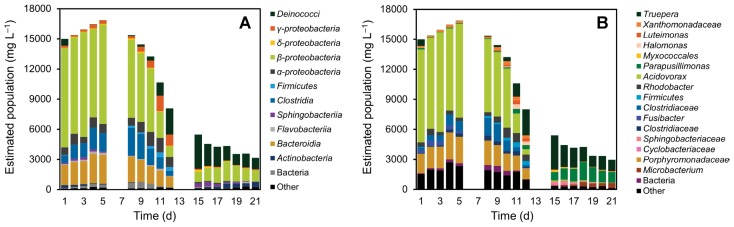
Structural changes in the microbial community. Relative distributions of sequences at the (A) class and (B) genus levels are shown. The microbial community structure in the activated sludge sample was analyzed by the high-throughput sequencing of 16S rRNA genes. Genomic DNA was extracted from 50 mg of activated sludge according to a direct lysis protocol (16) with minor modifications (14), and then purified and used as a template for PCR amplification. The V4 region of the 16S rRNA gene (approximately 250 bp) was amplified using the universal primers 515F and 806R (6). Both primers were modified to contain an Illumina adapter region, and the reverse primer contained a 12-bp barcode for multiplex sequencing (4). High-throughput sequencing was performed as described previously (2). An appropriate amount of the 16S rRNA gene fragments and an internal control (PhiX Control V3; Illumina, San Diego, CA, USA) were subjected to paired-end sequencing with a 500-cycle MiSeq reagent kit (Illumina) and MiSeq sequencer (Illumina). The removal of PhiX, low-quality (Phred value score [Q], <30), and chimeric sequences, and assembly of paired-end sequences were performed according to a previous study (10). Contaminating PhiX sequences in the libraries were detected using the Greengenes database (7) with Burrows-Wheeler Aligner, version 4.0.5 (13), and then removed by self-written scripts. Paired-end sequences were joined using a fastq-join tool in the ea-utils software package (https://code.google.com/p/ea-utils/downloads/list), version 1.1.2-301 (3). The joined sequences with Q scores ≥30 were collected using the QIIME software package, version 1.7.0 (5), and aligned using the mothur program, version 1.31.2 (21), after which the chimeric sequences were detected and excluded from the library. The sequences in each library were characterized phylogenetically using QIIME. Estimated population was determined by multiplying MLSS value by relative abundance of each taxonomic group.

**Table 1:** Ten operational taxonomic units (OTUs) with the highest fold change after a decrease in organic loading

Related species^1^	Accession	Identity	Increase^2^	Class^3^
*Truepera radiovictrix*	NR_074381.1	91%	63.00%	*Deinococci*
*Parapusillimonas granuli*	GQ422442.1	99%	14.20%	β (*Burkholderiales*)
*Aquiflexum balticum*	NR_025634.1	93%	3.40%	*Sphingobacteriia*
*Sphingobacterium* sp.	JQ514560.1	92%	2.60%	*Sphingobacteriia*
*Sorangium cellulosum*	HQ829402.1	95%	2.50%	δ (*Myxococcales*)
*Parvibaculum lavamentivorans*	NR_074262.1	99%	2.20%	α (*Rhizobiales*)
*Microbacterium arborescens*	KR259222.1	100%	1.00%	*Actinobacteria*
*Luteimonas marina*	NR_044458.1	100%	0.69%	γ (*Xanthomonadales*)
*Flavobacterium ummariense*	KF844048.1	96%	0.65%	*Flavobacteriia*
*Pseudidiomarina* sp.	GQ202579.1	100%	0.57%	γ (*Alteromonadales*)

1 The closest relatives of the OTUs were identified based on the results of a BLAST search (http://blast.ncbi.nlm.nih.gov/Blast.cgi) querying the 16S rRNA sequences against those in the DNA Data Bank of Japan (DDBJ) nucleotide sequence database (http://www.ddbj.nig.ac.jp/).

2 Increments in the relative abundance at day 15 relative to day 5 are shown.

3 The class (and order for *Proteobacteria*) of each OTU was predicted by QIIME. The symbols α, β, γ, and δ denote *α-*, *β-*, *γ-*, and *δ-proteobacteria*. Orders of proteobacterial OTUs are shown in parentheses.
